# Deep-Learning Assessed Muscular Hypodensity Independently Predicts Mortality in DLBCL Patients Younger Than 60 Years

**DOI:** 10.3390/cancers13184503

**Published:** 2021-09-07

**Authors:** Maxime Jullien, Benoit Tessoulin, Hervé Ghesquières, Lucie Oberic, Franck Morschhauser, Hervé Tilly, Vincent Ribrag, Thierry Lamy, Catherine Thieblemont, Bruno Villemagne, Rémy Gressin, Kamal Bouabdallah, Corinne Haioun, Gandhi Damaj, Luc-Matthieu Fornecker, Jean-Marc Schiano De Colella, Pierre Feugier, Olivier Hermine, Guillaume Cartron, Christophe Bonnet, Marc André, Clément Bailly, René-Olivier Casasnovas, Steven Le Gouill

**Affiliations:** 1 Department of Hematology, Nantes University Hospital, INSERM CRCINA Nantes-Angers, NeXT Université de Nantes, 44000 Nantes, France; maxime.jullien@chu-nantes.fr (M.J.); benoit.tessoulin@chu-nantes.fr (B.T.); 2 Department of Hematology, Hospices Civils de Lyon, Centre Hospitalier Lyon-Sud, Claude Bernard Lyon-1 University, 69310 Pierre Bénite, France; herve.ghesquieres@chu-lyon.fr; 3 Department of Hematology, IUC Toulouse Oncopole, 31000 Toulouse, France; oberic.lucie@iuct-oncopole.fr; 4 Department of Hematology, Univ. Lille, CHU Lille, EA 7365-GRITA-Groupe de Recherche sur les Formes Injectables et les Technologies Associées, 59000 Lille, France; franck.morschhauser@chru-lille.fr; 5 Department of Hematology, Centre H. Becquerel, 76000 Rouen, France; herve.tilly@chb.unicancer.fr; 6 Department of Hematology, Gustave Roussy, Université Paris-Saclay, 94800 Villejuif, France; ribrag@igr.fr; 7 Department of Hematology, University Hospital of Rennes, 35000 Rennes, France; thierry.lamy-de-la-chapelle@univ-rennes1.fr; 8 Department of Hematology, APHP, Hopital Saint Louis, Université Paris Diderot, 75011 Paris, France; catherine.thieblemont@aphp.fr; 9 Department of Hematology, Hopital Departemental de Vendée, 85000 La Roche sur Yon, France; bruno.villemagne@chd-vendee.fr; 10 Department of Hematology, CHU Grenoble, 38000 Grenoble, France; Rgressin@chu-grenoble.fr; 11 Department of Hematology, University Hospital of Bordeaux, F-33000 Bordeaux, France; krimo.bouabdallah@chu-bordeaux.fr; 12 Lymphoïd Malignancies Unit, Hôpital Henri Mondor, AP-HP, 94000 Créteil, France; corinne.haioun@aphp.fr; 13 Department of Hematology, Institut D’hématologie de Basse Normandie, 14000 Caen, France; damaj-gl@chu-caen.fr; 14 Department of Hematology, Institut de Cancérologie Strasbourg Europe (ICANS), University Hospital of Strasbourg, 67000 Strasbourg, France; lm.fornecker@icans.eu; 15 Department of Hematology, Institut P. Calmette, 13000 Marseille, France; schianojm@ipc.unicancer.fr; 16 Department of Hematology, University Hospital of Nancy, 54000 Nancy, France; p.feugier@chu-nancy.fr; 17 Department of Hematology, Hopital Necker, F-75015 Paris, France; olivier.hermine@aphp.fr; 18 Department of Clinical Hematology, University Hospital of Montpellier, UMR-CNRS 5535, 34000 Montpellier, France; g-cartron@chu-montpellier.fr; 19 Department of Hematology, CHU Liege, Liege University, 4000 Liege, Belgium; cbonnet@ulg.ac.be; 20 Department of Hematology, CHU UCL Namur, Université Catholique de Louvain, 5000 Namur, Belgium; marc.andre@unamur.be; 21 Department of Nuclear Medicine, University Hospital of Nantes, 44000 Nantes, France; clement.bailly@chu-nantes.fr; 22 Department of Hematology, University Hospital F. Mitterrand and Inserm UMR 1231, 21000 Dijon, France; olivier.casasnovas@chu-dijon.fr

**Keywords:** diffuse large B-cell lymphoma, muscle depletion, sarcopenia, muscle hypodensity, U-NET, convolutional neural network

## Abstract

**Simple Summary:**

Cachexia is a major cause of mortality in cancer patients and is characterized by a continuous skeletal muscle loss. Muscle depletion assessed by computed tomography (CT) is a predictive marker in solid tumors but has never been assessed in non-Hodgkin’s lymphoma. Despite software improvements, its measurement remains highly time-consuming and cannot be performed in clinical practice. We report the development of a CT segmentation algorithm based on convolutional neural networks. It automates the extraction of anthropometric data from pretherapeutic CT to assess precise body composition of young diffuse large B cell lymphoma (DLBCL) patients at the time of diagnosis. In this population, muscle hypodensity appears to be an independent risk factor for mortality, and can be estimated at diagnosis with this new tool.

**Abstract:**

Background. Muscle depletion (MD) assessed by computed tomography (CT) has been shown to be a predictive marker in solid tumors, but has not been assessed in non-Hodgkin’s lymphomas. Despite software improvements, MD measurement remains highly time-consuming and cannot be used in clinical practice. Methods. This study reports the development of a Deep-Learning automatic segmentation algorithm (DLASA) to measure MD, and investigate its predictive value in a cohort of 656 diffuse large B cell lymphoma (DLBCL) patients included in the GAINED phase III prospective trial (NCT01659099). Results. After training on a series of 190 patients, the DLASA achieved a Dice coefficient of 0.97 ± 0.03. In the cohort, the median skeletal muscle index was 50.2 cm^2^/m^2^ and median muscle attenuation (MA) was 36.1 Hounsfield units (HU). No impact of sarcopenia was found on either progression free survival (PFS) or overall survival (OS). Muscular hypodensity, defined as MA below the tenth percentile according to sex, was associated with a lower OS and PFS, respectively (HR = 2.80 (95% CI 1.58–4.95), *p* < 0.001, and HR = 2.22 (95% CI 1.43–3.45), *p* < 0.001). Muscular hypodensity appears to be an independent risk factor for mortality in DLBCL and because of DLASA can be estimated in routine practice.

## 1. Introduction

Cachexia is a major cause of mortality in solid tumors [1]. Involuntary weight loss greater than 5% was recognized 40 years ago as a mortality predictor in several cancers, including non-Hodgkin’s lymphomas (NHLs) [2]. Cachexia is a multifactorial syndrome characterized by a continuous loss of skeletal muscle mass with or without the loss of fat mass, due to a negative protein and energy balance resulting from a variable combination of abnormal metabolism and reduced dietary intake [3]. Muscle depletion (MD) is described quantitatively by muscle size reduction, and qualitatively by an increase in the proportion of inter- and intramuscular fat. A computed tomography (CT) scan has become a reference for MD assessment [4]: an analysis of a CT slice at the level of the 3rd lumbar vertebra (L3) accurately predicts the entire body’s fat and lean body mass [5], enabling the calculation of a skeletal mass index (SMI) by estimating muscle size [3], and muscle attenuation (MA), which leads to muscle fat content [6]. However, current methods of MD estimation require manual muscle definition of CT slices and have to be performed by a trained operator using dedicated software. These constraints limit its use in daily practice [7]. A solution was found in the automation of segmentation by using Deep Learning. This machine learning allows computer models, using artificial neural networks, to autonomously learn abstract representations from large amounts of data. For the learning process, the data are provided to the algorithm in the form of “ground truths”, corresponding to an ideal expected result. Among these models, convolutional neural networks (CNN) led to major advances in image and video recognition [8]. U-Net, launched in 2015, is one such CNN developed for segmentation of biomedical images [9]. U-Net architectures have since been used in several fields including radiotherapy [10], anatomical pathology [11] and imaging [12,13]. More recently, several teams [14,15,16,17] have published the feasibility of muscle segmentation on routine CT L3 slices using U-Net.

The prognostic value of MA and SMI has largely been reported in oncology populations [18,19,20,21,22], but as far as hematology is concerned only a few retrospective studies have been reported, mostly in geriatric populations [23,24,25]. Yet, identification of such markers of fragility could have a major therapeutic impact in younger patients presenting with no comorbidity and considered eligible for intensive chemotherapy.

We report here on the development of a Deep Learning automatic segmentation algorithm (DLASA) based on low-resolution, non-injected CT scans extracted from lymphoma patients’ pretherapeutic positron emission tomography (PET)–CT scans, and aimed at assessing their body composition. This algorithm enables rapid assessment of SMI and MA, as well as visceral and subcutaneous fat tissue from the L3 slice. It was applied to review CT scans of young DLBCL patients undergoing intensive first-line treatment in the prospective phase III GAINED study [26]. The objective was to determine whether these parameters could be a predictive factor.

## 2. Materials and Methods

### 2.1. Patients Selection

To increase diversity and reduce overfitting, the training set consisted of 190 patients included in 2 prospective trials of the Lymphoma Study Association (LYSA) group (GAINED and LyMa trials). In brief, GAINED (NCT01659099) was a prospective phase III trial carried out from 2013 to 2015 [26] that compared Obinutuzumab vs. Rituximab (R) in combination with CHOP (cyclophosphamide, doxorubicin, vincristine, and prednisone) or ACBVP (*idem* with bleomycin and vindesine replacing vincristine) chemotherapy in newly diagnosed untreated DLBCL patients under 60 years of age. The other main inclusion criteria were an age-adjusted International Prognostic Index (aaIPI) ≥1; eligibility for autologous stem cell transplantation (ASCT); life expectancy ≥3 months; and normal liver, renal and hematological function unless abnormalities were related to DLBCL. The therapeutic strategy was guided by an interim metabolic evaluation by FDG–PET (PET) after cycle 2 (PET2) and 4 (PET4), leading to a therapeutic intensification with (ASCT) in case of insufficient response. The results showed no difference between the 2 antibodies. LyMa (NCT00921414) was a prospective trial which evaluated rituximab maintenance following ASCT in patients with previously untreated mantle cell lymphoma [27]. All included patients performed a baseline PET-CT prior to the beginning of treatment. A total of 100 patients from LyMa and 90 randomly selected patients from GAINED were included in the training set. The algorithm’s training validation process was performed on a testing set including 49 patients randomly selected from GAINED and different from the training cohort. The exploratory set consisted of the 670 patients included in the GAINED trial.

### 2.2. Ground Truth Generation

Matlab^®^ software (MathWorks, version R2019b, Natick, MA, USA) was used to train and test image preparation, ground-truth generation and the neural network. Generation of ground truth for DLASA training and testing purpose was carried out on the 239 patients mentioned above. For each of them, L3 vertebrae CT slices were manually extracted from the baseline PET–CT scans. Ground truths were generated by a trained operator (author MJ, a medical doctor, after training for manual segmentation of muscle and fat), who used an algorithm requiring manual contouring of regions of interests (ROIs), which corresponded to 10 different muscle groups (as reported by Burns et al.) and to visceral and subcutaneous adipose tissues. Automatic pixel selection, using Hounsfield density thresholds of (−29 to +150 HU) for muscle and (−190 to −30 HU) for fat, was then performed.

### 2.3. DLASA Trainings and Validation

For each of the 12 ROIs, a distinct U-Net neural network (6-layer depth) was created and trained using Matlab DeepLearning Toolbox extension. The training was performed on a NVIDIA GTX 970 (4 GB DDR) CPU. The initial learning rate was set at 10^−3^, using the Adam optimizer, with a maximum of 300 epochs. The output of all 10 CNN trained for automatic segmentation of muscle groups, was pooled to constitute the muscle ROI. The DLASA was then confirmed on the 49 slices of the testing set. The automatic segmentation performance was evaluated using Dice’s formula as previously published [14,28]:

$$Dice=\frac{2\left(A\;\cap \;M\right)}{A+M}$$

where *A* corresponds to the automatic segmentation matrix, *M* the manual segmentation matrix, and ∩ the intersection.

### 2.4. Evaluation of Body Composition in a Prospective Cohort Using the Algorithm

Image acquisition parameters such as slice thickness (ST), the use of a dose saving system, or adaptation of the tube current to the patient’s BMI were performed according to the practices of the different centers. The validated DLASA was applied to baseline PET–CT of all patients in the exploratory set after a manual L3 slice selection. The muscle ROI corresponding surface area was expressed in cm^2^. The SMI (cm^2^/m^2^) was calculated by dividing the muscle area obtained in L3 by the patient’s height in meters squared. The threshold for sarcopenia was 55 cm^2^/m^2^ for men and 39 cm^2^/m^2^ for women [3]. Similarly, the fat mass index was defined as the fat area divided by the height squared, marking a distinction between the visceral (VAT) and subcutaneous (SAT) fat mass index. Finally, the MA was calculated by averaging the density of each pixel belonging to the muscle ROI, in HU. In the absence of a consensual published threshold, muscular hypodensity was defined as a MA value below the tenth percentile of the cohort for each gender. The selected cut-off was 26.7 HU for males and 23.1 HU for females (*n* = 65 patients).

### 2.5. Outcomes and Statistics

Responses to chemotherapy were assessed metabolically in the exploratory set by PET2 and PET4, as previously described [26]. Overall survival (OS) was defined as the time interval between the start of treatment and death from any cause. Progression-Free Survival (PFS) was defined as the time interval between treatment initiation and progression or relapse (according to Cheson 2007 criteria [29]) or death from any cause. Statistical analyses were performed on R (version 4.0.3). Survival functions were calculated using Kaplan–Meier estimates, and a comparison between categories was made using the log-rank test. Relapse and non-relapse mortality (NRM) were estimated using the cumulative incidence method. Characteristics of populations were compared by using Χ^2^ test for discrete variables and Student’s *t* test for continuous variables. Multivariable analyses were performed by using Cox proportional hazards models. A ROC curve analysis was used to determine cut-off values for the different variables of various outcomes. The area under the ROC curve (AUC) was used to estimate the discriminant power of a variable, with an AUC of 0.5 indicating zero discriminant power and an AUC of 1 indicating perfect discrimination. A threshold of 0.7 was used to identify variables of interest as previously published [22].

## 3. Results

### 3.1. Segmentation Algorithm Training and Validation

**Figure 1:**
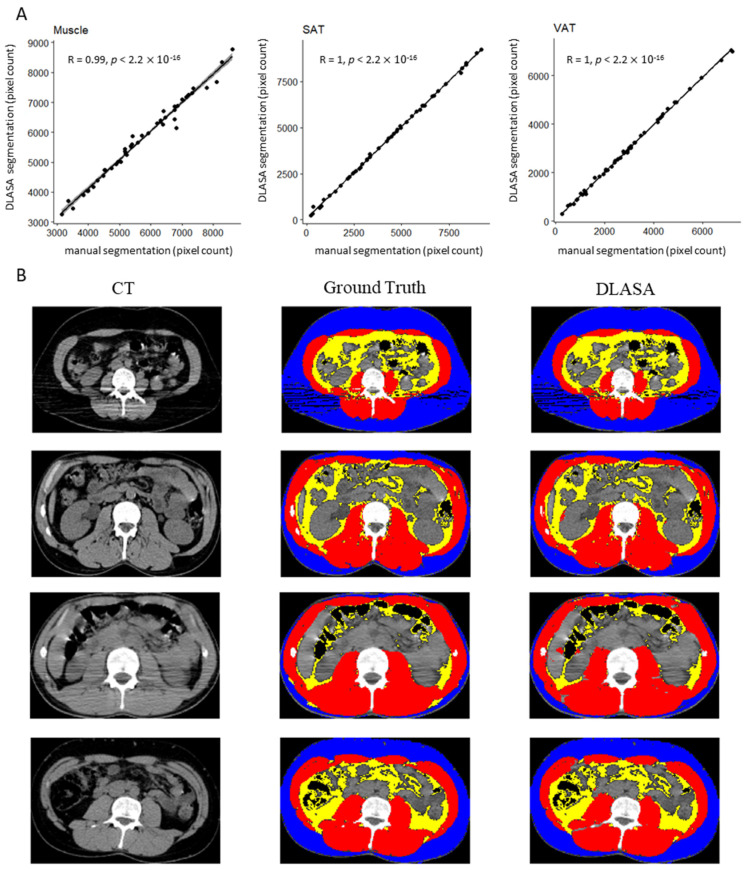
(**A**) Correlation between manual segmentation and DLASA segmentation. (**B**) Visual rendering of manual (ground truth) and automated (DLASA) segmentation. DLASA: Deep Learning automated segmentation algorithm. SAT: subcutaneous adipose tissue (blue). VAT: visceral adipose tissue (yellow). Muscle region of interest (red).

The DLASA was trained on the 190 patients from the training set and validated on the 49 patients from the testing set. Patient characteristics in the training, testing and exploratory sets are reported in Table S1. Patients in the training set were older than those in the testing and exploratory sets (54 vs. 48, respectively, *p* = 0.003, *p* < 10^−9^). The characteristics of patients in the testing and exploratory sets were similar. The validation of the trained model in the testing set showed a good performance without an overfitting issue: Dice coefficients for detection of total abdominal muscle, subcutaneous adipose tissue and visceral adipose tissue were, respectively, 0.97 ± 0.03, 0.97 ± 0.06 and 0.97 ± 0.03. The mean absolute differences between manual (ground truth) and DLASA measurements for SMI, MA, SAT and VAT are reported in Table 1. Regarding pixel counts, the correlation with manual measurement was close to 1 for all three ROIs, as shown in Figure 1A. Examples of the segmentation’s visual rendering are presented in Figure 1B.

### 3.2. Anthropometric Evaluation in the Exploratory Set

Among the 670 patients of the GAINED study, 667 had a pretherapeutic PET–CT available. Eleven were excluded because of major PET–CT artefacts in the L3 region. Anthropometric characteristics of the 656 patients in the exploratory set are reported in Table 2. Median age at diagnosis was 48 years (IQR 38–55). BMI was slightly higher in men (24.6 vs. 23.4 kg/m^2^, *p* = 0.02) with no difference in percentage of obesity (*p* = 0.41).

SMI and MA were measured using DLASA. SMI was higher in men (55.5 vs. 44.9 kg/m^2^), but more patients were considered sarcopenic in the male population (48.8 vs. 15.9%, *p* < 10^−15^). SMI did not fluctuate with age, regardless of gender, and was correlated with weight (Supplementary Figure S1). MA was higher in males (37.8 vs. 34.2 HU, *p* < 10^−6^), decreased with age and BMI regardless of gender, and was independent of SMI (Supplementary Figure S2). Of note, ST varied from 1 to 6.5 mm, with a median of 3 mm (IQR: 2.5–3.75 mm). No variation was observed for SMI for ST < or ≥3 mm (*p* = 0.26). MA appeared to vary slightly with ST: median 36.7 vs. 35.2 HU (*p* = 0.01), but proportion of patients estimated with muscle hypodensity did not vary significantly (*p* = 0.21). No information was available regarding the use of a dose-saving system.

### 3.3. Outcome According to Anthropometric Evaluation

Of the 656 patients in the exploratory set, 639 (97.4%) were evaluated for PET2, and 615 (93.8%) for PET 4. Responses according to obesity, muscular hypodensity and sarcopenia are reported in Table 3. Among patients evaluated, no impact on PET2 and PET4 response was observed for these different parameters. It should be noted that compared to the control population, a larger proportion of patients with muscle hypodensity did not undergo a metabolic evaluation: after 2 courses: 7.7 vs. 2.0%, *p* = 0.02, after 4 courses: 15.4 vs. 5.2%, *p* = 0.004. Such a difference was not observed in obese or sarcopenic patients.

The median follow-up for the entire cohort was 36.6 months (IQR 29.9–45.1). In univariate analysis (Table 4), obesity (BMI > 30 kg/m^2^) had a negative impact on OS: HR 2.30 (95% CI 1.36–3.87), 36 m OS 80.2 vs. 89.9%, *p* = 0.002; and PFS: HR 1.84 (95% CI 1.22–2.77), 36 m PFS 66.6 vs. 80.9%, *p* = 0.004. BMI < 18.5 kg/m^2^ had no impact on survival. Muscular hypodensity had a negative impact on OS: HR 2.80 (95% CI 1.58–4.95), 36 m OS 75.9 vs. 89.9%, *p* < 0.001; and PFS: HR 2.22 (95% CI 1.43–3.45), 36 m PFS 61.4 vs. 80.8%, *p* < 0.001. Sarcopenia had no impact on OS or PFS. It should be noted that SMI had no impact even considering various cut-offs (median, twentieth and tenth percentile by gender, data not shown). Age had a deleterious impact only on OS: HR 1.03 (95% CI 1.01–1.05), *p* = 0.02, but not on PFS (*p* = 0.19). aaIPI score had a prognostic value on OS and PFS. As previously reported [26], the treatment arm had no impact on survivals.

**Figure 2:**
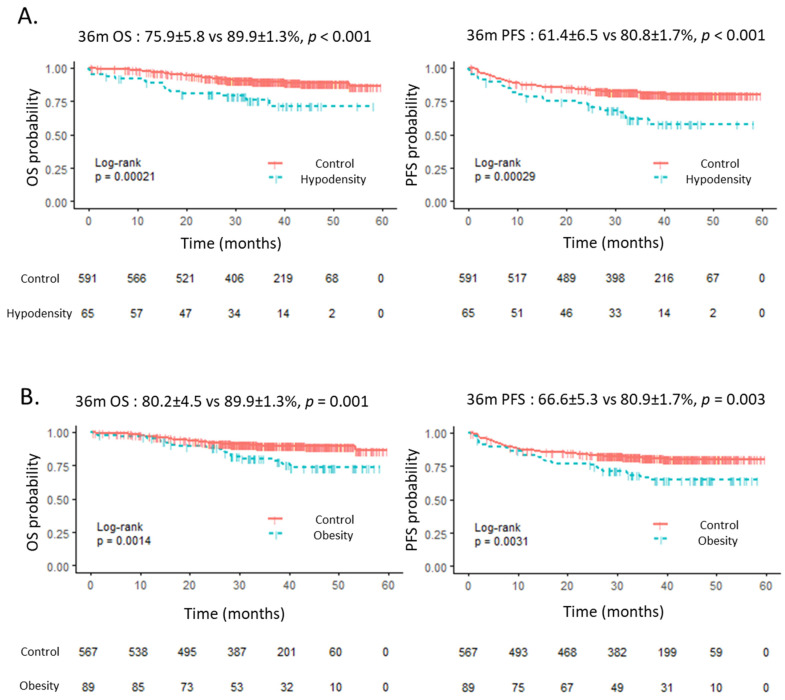
OS and PFS according to muscle hypodensity (**A**) and obesity (**B**). OS: overall survival. PFS: progression-free survival.

The multivariate analysis including the identified anthropometric variables, age and aaIPI, as covariates, is reported in Table 5. When taking into account the correlation between BMI and MA, obesity and muscular hypodensity remained statistically associated with OS and PFS: for muscular hypodensity HR = 2.22 (95% CI: 1.04–4.70, *p* = 0.04) for OS, and HR = 2.06 (95% CI: 1.16–3.67, *p* = 0.01) for PFS. Figure 2 illustrates the impact of muscular hypodensity and obesity on OS and PFS.

Considering death and relapse as two competing risks, cumulative incidence analyses suggested that muscular hypodensity and obesity were statistically related to a higher incidence of NRM: respectively, 36 m NRM 12.7 ± 1.8% vs. 2.0 ± 0.0; Gray test *p* < 0.001, and 8.1 ± 0.9% vs. 2.2 ± 0.0, *p* < 0.001, with no difference in relapse (*p* = 0.40 and *p* = 0.11, respectively). Supplementary Figure S3. During the follow up period, 73 deaths occurred. Mortality rates were, respectively, 15/65 (23.1%) in patients with muscular hypodensity vs. 58/591 (9.8%) for controls (*p* = 0.003). The causes of death are summarized in Supplementary Table S2. The major causes in both groups were lymphoma (respectively, 9.2 vs. 6.6%, *p* = 0.43) and toxicity (6.2 vs. 1.9%, *p* = 0.05).

## 4. Discussion

In the present study we reported the development of a DLASA that enabled automatic extraction of MA and SMI from L3 slices of a pretherapeutic PET–CT. Its accuracy was comparable to that of other previously published neural networks (Table S3). It achieved a high Dice despite its training on a relatively small number of non-injected CT slices, which made muscle surface discrimination even more challenging. This result could be explained, in part, by the independent training of a different CNN for each muscle group. It is likely that a better Dice coefficient could be achieved by training the CNN on a larger number of slices. The main difference between the present DLASA and the above-mentioned CNN lay in its ability to segment visceral and subcutaneous adipose tissue at the same time with extreme accuracy. Finally, this DLASA was trained on low-resolution, non-injected L3 CT slices, making it suitable for routine non-injected PET and CT analysis. The expected automatic operation is effective with a result rendered as in image format and a complete accounting of the parameters in about 6 s from the DICOM file. The present study was not focused specifically on the automatic selection of the L3 slice. As this manual selection step remains time consuming, its automation using another CNN, as demonstrated by Belharbi et al. [30], would be interesting.

The second part of our work investigated the impact of muscular hypodensity and sarcopenia on the outcome of young DLBCL patients receiving frontline therapy in the GAINED study. Because of the retrospective nature of this CT slice analysis, image acquisition parameters such as ST or adaptation of the tube current to the patient’s corpulence were performed according to the practices of the different centers, which may be one of the limitations of this study. However, no relevant impact of ST on muscular hypodensity estimation nor SMI measure was found in this series. Several studies have reported the impact of CT scan image acquisition parameters on body composition analysis, with contradictory results regarding the effects of ST and radiation dose reduction on SMI and MA measure [31,32]. In both papers, intravenous contrast material has however a strong effect on those measures. The use of an exclusively non-contrast pool of CT scans in the present study allows to eliminate this bias in MA and SMI estimation. Future prospective studies evaluating these different parameters should focus on standardizing the acquisition parameters to avoid further bias.

Several studies described the deleterious impact of sarcopenia, which was estimated by measuring SMI [18,19,20,21,22,23]. There currently exists no consensual definition of sarcopenia using this method, as evidenced by the use of different thresholds. Lee et al. [18] and Cho et al. [19] used thresholds corresponding to Korean standards; Kim et al. [20] used the thresholds recommended by Fearon et al. [3]; Daly et al. [21] and Martin et al. [22] recommended gender-specific and BMI thresholds; and Lanic et al. [23] used a gender-specific threshold corresponding to a SMI below the 20th percentile of their population. The high prevalence of sarcopenia in the male population of the study (48%) seemed exceptionally high, especially given the fact that these patients were selected to enter a first-line clinical trial, which generally leads to a selection bias for less severe patients in better general condition. The hypothesis of a wasting effect due to the lymphoma seems unlikely here because the rate of sarcopenia was much lower (16%) in the female population of this cohort. This anomaly was rather probably related to the application of cut-offs not adapted to the European population. Unlike the studies previously mentioned, no impact of sarcopenia was found in our cohort, whatever the SMI threshold used. The impact of sarcopenia measured by SMI may be overestimated in the literature because of publication bias. Moreover, it was described in a geriatric context [23] and, in solid oncology, mostly in advanced cancers [18,20,21] or in neoplasia directly affecting nutritional status, such as ENT cancers [19]. Herein, the population under study was young, without co-morbidities, transplant eligible, and included in a prospective phase III trial for a previously untreated DLBCL. Finally, as DLBCL is an aggressive disease, it is likely that the response to treatment statistically overwhelmed the other variables. All these reasons may explain the lack of impact on SMI found in the present study.

On the other hand, pretherapeutic muscular hypodensity has a deleterious impact on both OS and PFS, independent of aaIPI and obesity. A similar observation was reported in a monocentric retrospective study with DLBCL patients who were more heterogeneous and older than in the present study [25]. Therefore, it is likely that this negative impact can be found in all age groups in DLBCL patients. The deleterious effect of muscle hypodensity has been described in the context of several solid cancers [22,33,34], but the physiological basis behind this effect on survival is yet unknown. Muscle hypodensity is considered to be related to excess intramuscular fat [6], but MA values can also decrease with systemic edema. In this series, the deleterious effect of muscle hypodensity on survival was not related to a lower response rate to chemotherapy or to a higher rate of disease relapse but to an increased non-relapse mortality with a trend towards a higher rate of toxicity-related deaths. The present algorithm enabled the identification of this mortality risk factor at the time of diagnosis. The impact of corrective measures for muscular hypodensity, such as coaching by a nutritionist and physical training, deserves to be further explored.

## 5. Conclusions

We reported the development of a CT segmentation algorithm based on CNN. It automated the extraction of anthropometric data from pretherapeutic L3 slices from CT scan to assess precise body composition of DLBCL patients at the time of diagnosis. Unlike the previous findings of many studies in solid oncology, sarcopenia (estimated from SMI) did not have a deleterious effect on young DLBCL patients receiving intensive frontline therapy in the setting of a clinical trial. However, in this population, muscle hypodensity appeared to be a risk factor for mortality, independent of obesity, treatment or aaIPI in DLBCL and can be estimated at diagnosis with this new tool.

## Figures and Tables

**Table 1:** Algorithm performance for CT segmentation.

Variable	Ground Truth	DLASA	*p* Value
SMI (cm^2^/m^2^), median (IQR)	48.6 (44.5–56.3)	49.1 (44.8–57.4)	0.65
Abs difference (mean ± SD)	1.18 ± 1.2		
MA (HU), median (IQR)	38.9 (33.8–43.0)	38.6 (33.4–42.5)	0.80
Abs difference, HU (mean ± SD)	0.55 ± 0.4		
SAT (cm^2^/m^2^), median (IQR)	35.2 (21.5–61.5)	35.3 (22.4–62.0)	0.84
Abs difference (mean ± SD)	0.71 ± 0.6		
VAT (cm^2^/m^2^), median (IQR)	22.9 (12.0–40.5)	22.9 (12.3–40.4)	0.97
Abs difference (mean ± SD)	0.47 ± 0.6		
Dice (mean ± SD)			
muscle	0.97 ± 0.03		
SAT	0.97 ± 0.06		
VAT	0.97 ± 0.03		

DLASA: Deep Learning automated segmentation algorithm. SMI: skeletal muscle index. MA: muscle attenuation. SAT: subcutaneous adipose tissue index. VAT: visceral adipose tissue index. Abs: absolute. Absolute differences and Dice are given as mean ± SD. SMI, MA, SAT, VAT are given as median (interquartile range).

**Table 2:** Patient characteristics at baseline.

	*n* = 656		
	Male	Female	*p*
*n* (%)	367 (55.9)	289 (44.1)	
Age (years)	49 (40–56)	47 (36–54)	0.01
BMI (kg/m^2^)	24.6 (22.2–27.8)	23.4 (20.8–27.3)	0.02
<18.5, *n* (%)	11 (3.0)	23 (8.0)	0.01
>30, *n* (%)	45 (12.3)	44 (15.2)	0.41
SMI (cm^2^/m^2^)	55.5 (50.6–61.4)	44.9 (40.9–48.1)	<10^−15^
sarcopenia, *n* (%)	179 (48.8)	46 (15.9)	<10^−15^
MA (HU)	37.8 (32.6–43.6)	34.2 (28.5–40.0)	<10^−6^
muscular hypodensity, *n* (%)	38 (10.3)	27 (9.3)	0.77
aaIPI			
low, *n*(%)	1 (0.3)	1 (0.3)	1
low-int, *n*(%)	147 (40.1)	130 (45.0)	0.23
high-int, *n*(%)	173 (47.1)	130 (45.0)	0.64
high, *n*(%)	46 (12.5)	28 (9.7)	0.31

BMI: body-mass index. SMI: skeletal muscle index. MA: muscle attenuation. aaIPI: age-adjusted international prognostic index. Unless otherwise stated, numbers are given as median (interquartile range). SMI and MA were measured using DLASA.

**Table 3:** PET2 and PET4 results according to obesity, sarcopenia, and muscular hypodensity.

		Obese	Control	*p*	Sarcopenia	Control	*p*	Muscular Hypodensity	Control	*p*
PET 2	Neg	61 (70.1%)	391 (70.8%)	0.99	149 (69.3%)	303 (71.5%)	0.63	45 (75.0%)	407 (70.3%)	0.54
	Pos	26 (29.9%)	161 (29.2%)		66 (30.7%)	121 (28.5%)		15 (25.0%)	172 (29.7%)	
PET 4	Neg	66 (77.6%)	446 (84.2%)	0.18	171 (82.2%)	341 (83.8%)	0.70	48 (87.3%)	464 (82.9%)	0.52
	Pos	19 (22.4%)	84 (15.8%)		37 (17.8%)	66 (16.2%)		7 (12.7%)	96 (17.1%)	

Numbers are given as *n* (%). Neg: negative according to the GAINED response criteria. Pos: positive.

**Table 4:** Univariate analysis on OS and PFS.

	OS			PFS		
Variable	HR	95% CI	*p*	HR	95% CI	*p*
Obesity (BMI > 30 kg/m^2^)	2.30	1.36–3.87	0.002	1.84	1.22–2.77	0.004
Underweightness (BMI < 18.5 kg/m^2^)	1.76	0.76–4.06	0.19	1.75	0.94–3.24	0.08
Muscle hypodensity	2.80	1.58–4.95	<0.001	2.22	1.43–3.45	<0.001
Sarcopenia	1.20	0.74–1.93	0.46	1.27	0.90–1.80	0.18
aaIPI	1.88	1.35–2.63	<0.001	1.48	1.16–1.90	0.002
age	1.03	1.01–1.05	0.02	1.01	0.99–1.03	0.19
Rituximab vs. Obinutuzumab	1.08	0.68–1.73	0.74	0.99	0.71–1.40	0.96
CHOP vs. ACVBP	1.11	0.70–1.78	0.65	1.16	0.82–1.63	0.41

OS: overall survival. PFS: progression-free survival. BMI: body-mass index. aaIPI: age-adjusted international prognostic index. CHOP: cyclophosphamide, doxorubicin, vincristine, and prednisone. ACBVP: cyclophosphamide, doxorubicin, bleomycin, vindesine and prednisone.

**Table 5:** Multivariate analysis on OS and PFS.

	OS			PFS		
Variable	HR	95% CI	*p*	HR	95% CI	*p*
Obesity	2.18	1.18–4.06	0.01	1.81	1.11–2.94	0.02
Muscle hypodensity	2.22	1.04–4.70	0.04	2.06	1.16–3.67	0.01
aaIPI	1.73	1.23–2.44	0.002	1.40	1.09–1.80	0.009
age	1.02	0.99–1.04	0.14	1	0.99–1.02	0.72

OS: overall survival. PFS: progression-free survival.

## Data Availability

Request for access to the study data can be asked by email to the corresponding authors.

## Supplementary Materials

The following are available online at https://www.mdpi.com/article/10.3390/cancers13184503/s1, Table S1: Patient characteristics in the training, testing and exploratory sets. Table S2: Causes of death in the exploratory set. Table S3: Performances of previously published neural networks. Figure S1: Correlations of SMI with age and BMI. Figure S2: Correlation of MA with age, SMI and BMI. Figure S3: Cumulative incidence of death or relapse according to muscular hypodensity or obesity.
