# Supplementary material for: Deep-Learning Assessed Muscular Hypodensity Independently Predicts Mortality in DLBCL Patients Younger Than 60 Years
Source: Cancers (Basel). 2021 Sep 7;13(18):4503. doi: 10.3390/cancers13184503 (PMC8466314; doi:10.3390/cancers13184503)
Supplement: Supplementary file 1 [file cancers-13-04503-s001.zip › cancers-1285435-supplementary.pdf]

Supplementary data

Table S1. Patients characteristic in the training, testing and exploratory sets.

	Training set	Testing set	Exploratory set
n	190	49	656
age (years)	54.0 (46.0 - 59.0)	48.0 (42.0 - 56.0)	48.0 (37.7 - 55.0)
female, *n* (%)	67 (35.3%)	16 (32.7%)	289 (44.1%)
neoplasia			
DLBCL, *n*(%)	90 (47.4%)	49 (100%)	656 (100%)
MCL, *n*(%)	100 (52.6%)	-	-
BMI (kg/m²)	25.6 (22.3 - 28.1)	23.3 (21.0 - 25.7)	24.1 (21.8 - 27.7)
SMI (cm²/m²)	52.5 (45.6 - 58.7)	49.1 (44.8 - 57.4)	50.3 (44.6 - 57.2)
MA (HU)	34.9 (29.9 - 39.9)	38.6 (33.4 - 42.5)	36.1 (30.4 - 42.0)

DLBCL diffuse large B cell lymphoma; MCL mantle cell lymphoma; BMI body mass index; SMI skeletal muscle index; MA muscle attenuation. Unless otherwise stated, numbers are given as median (IQR).

Table S2. Causes of death in the exploratory set.

Cause	Muscular hypodensity (n=65)		Control (n=591)		*p*
Lymphoma	6	(9.2%)	39	(6.6%)	0.43
Toxicity	4	(6.2%)	11	(1.9%)	0.05
Concurrent illness	3	(4.6%)	5	(0.8%)	0.03
Other reason	1	(1.5%)	3	(0.5%)	0.34
Unknown	1	(1.5%)	0	(0.0%)	0.10
**Total**	**15**	**(23.1%)**	**58**	**(9.8%)**	**0.003**

Numbers are given as n(%).

Table S3. Performances of previously published neural networks.

Study	Training sample, n	Validation sample, n	Dice
Burns et al.	51	51	0.94 $\pm$ 0.03
Lee et al.	250	150	0.93 $\pm$ 0.02
Edwards et al.	682	137	0.92 $\pm$ 0.02
Dabiri et al.	3774	1327	0.99 $\pm$ 0.01
**Present study**	**190**	**49**	**0.97 $\pm$ 0.03**

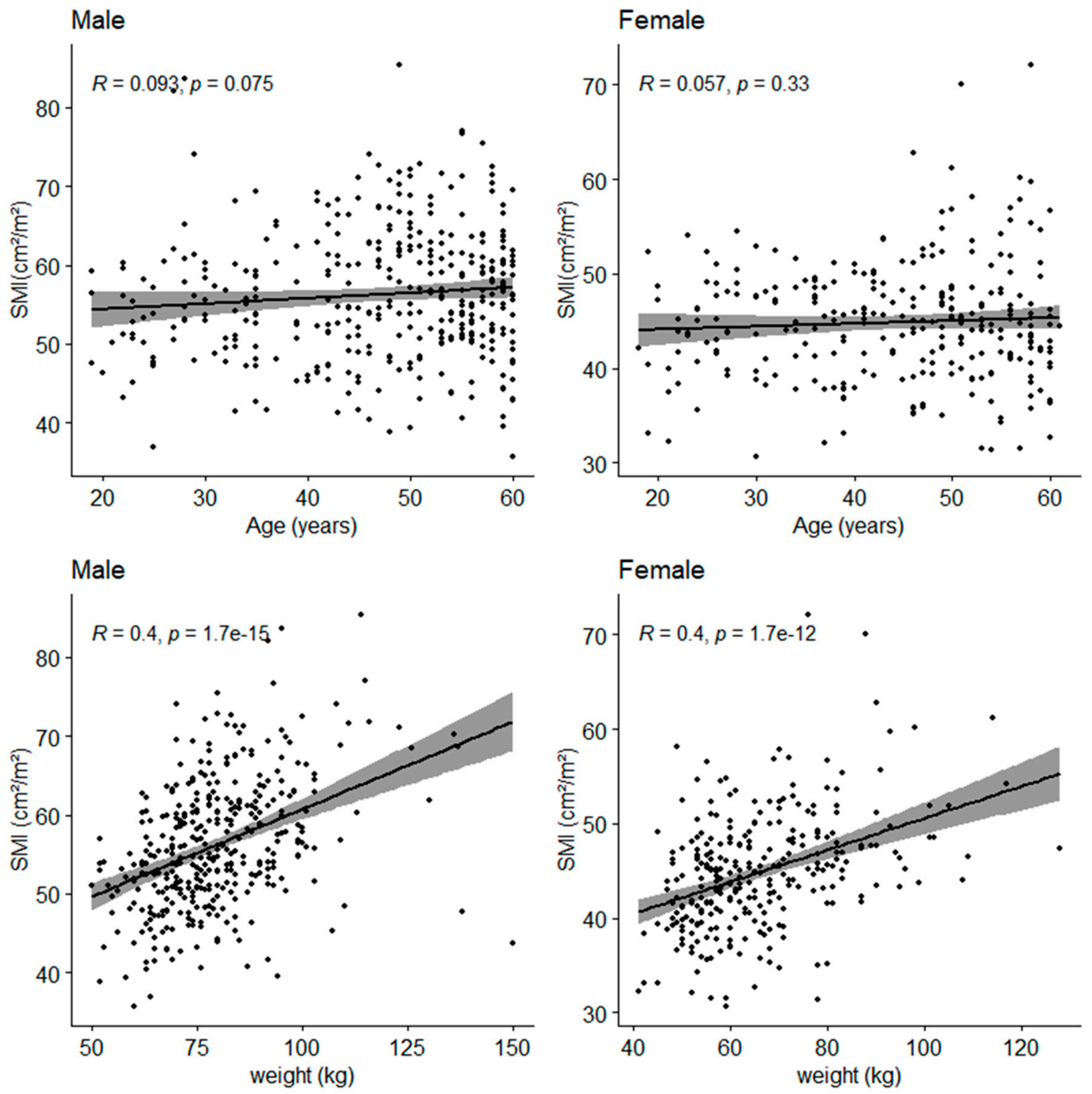

Figure S1. Correlations of SMI with age and weight.

SMI skeletal muscle index; body mass index.

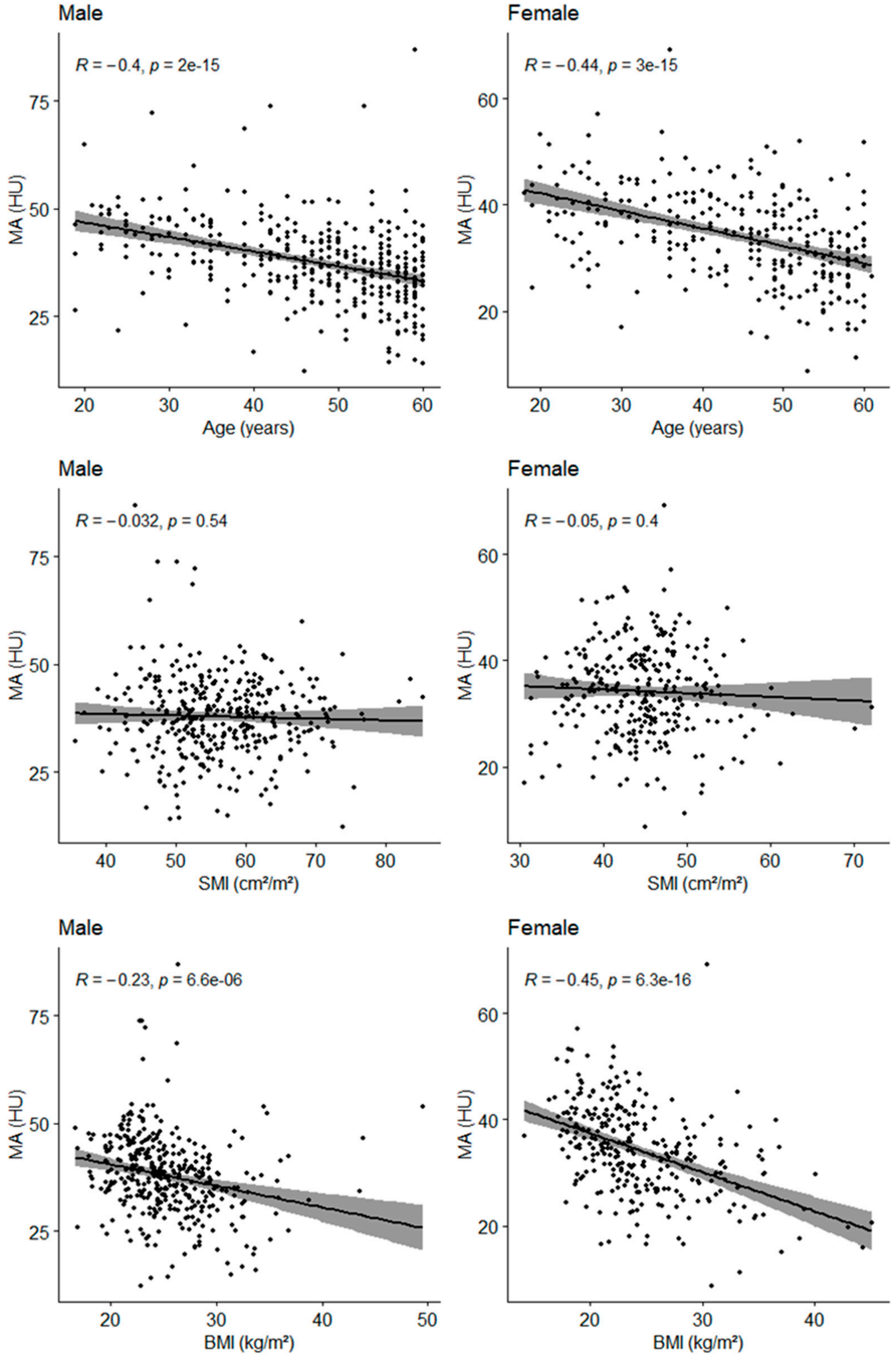

Figure S2. Correlation of MA with age, SMI and BMI

MA muscle attenuation; SMI skeletal muscle index; BMI body mass index.

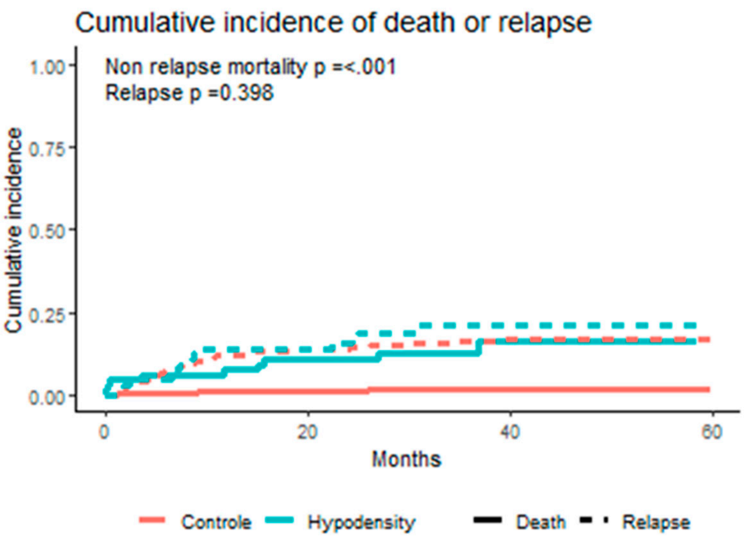

36M NRM : 12.7±1.8% vs 2.0±0.0, p<0.001

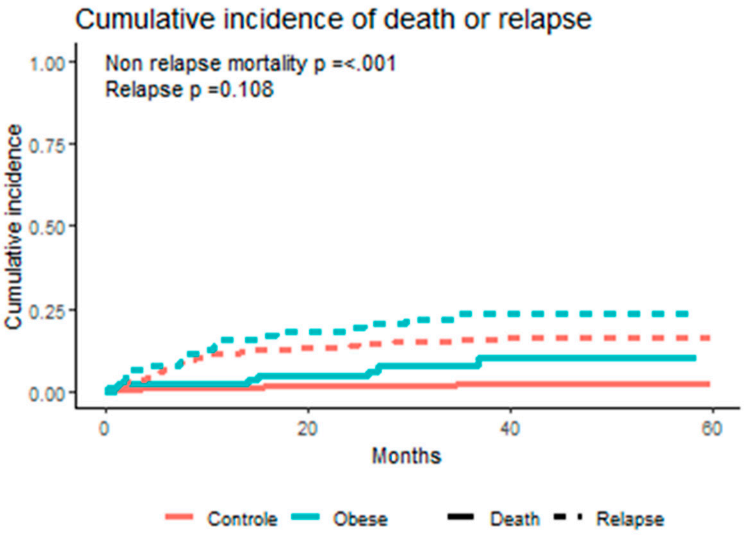

36M NRM : 8.1±0.9% vs 2.2±0.0, p<0.001

Figure S3. Cumulative incidence of death or relapse according to muscular hypodensity or obesity.

NRM non relapse mortality.
